# Assessment of Postoperative Opioid Prescriptions Before and After Implementation of a Mandatory Prescription Drug Monitoring Program

**DOI:** 10.1001/jamahealthforum.2021.2924

**Published:** 2021-10-01

**Authors:** Rivfka Shenoy, Zachary Wagner, Allison Kirkegaard, Robert J. Romanelli, Satish Mudiganti, Louis Mariano, Meghan Martinez, Kyle Zanocco, Katherine E. Watkins

**Affiliations:** 1 David Geffen School of Medicine, Department of Surgery, University of California, Los Angeles, Los Angeles; 2 Veterans Health Administration, Greater Los Angeles Healthcare System, Los Angeles, California; 3 National Clinician Scholars Program, University of California, Los Angeles, Los Angeles; 4 RAND Corporation, Santa Monica, California; 5 Center for Health Systems Research, Division of Research, Development and Dissemination, Sutter Health, Walnut Creek, California; 6 Center for Health Systems Research, Palo Alto Medical Foundation Research Institute, Sutter Health, Palo Alto, California

## Abstract

**Importance:**

Legislation mandating consultation with a prescription drug monitoring program (PDMP) was implemented in California on October 2, 2018. This mandate requires PDMP consultation before prescribing a controlled substance and integrates electronic health record (EHR)–based alerts; prescribers are exempt from the mandate if they prescribe no more than a 5-day postoperative opioid supply. Although previous studies have examined the consequences of mandated PDMP consultation, few have specifically analyzed changes in postoperative opioid prescribing after mandate implementation.

**Objective:**

To examine whether the implementation of mandatory PDMP consultation with concurrent EHR-based alerts was associated with changes in postoperative opioid quantities prescribed at discharge.

**Design, Setting, and Participants:**

This cross-sectional study performed an interrupted time series analysis of opioid prescribing patterns within a large health care system (Sutter Health) in northern California between January 1, 2015, and February 1, 2020. A total of 93 760 adult patients who received an opioid prescription at discharge after undergoing general, obstetric and gynecologic (obstetric/gynecologic), or orthopedic surgery were included.

**Exposures:**

Mandatory PDMP consultation before opioid prescribing, with concurrent integration of an EHR alert. Prescribers are exempt from this mandate if prescribing no more than a 5-day opioid supply postoperatively.

**Main Outcomes and Measures:**

The primary outcome was the total quantity of opioid medications (morphine milligram equivalents [MMEs] and number of opioid tablets) prescribed at discharge before and after implementation of the PDMP mandate, with separate analyses by surgical specialty (general, obstetric/gynecologic, and orthopedic) and most common surgical procedure within each specialty (laparoscopic cholecystectomy, cesarean delivery, and knee arthroscopy). The secondary outcome was the proportion of prescriptions with a duration of longer than 5 days.

**Results:**

Of 93 760 patients (mean [SD] age, 46.7 [17.6] years; 67.9% female) who received an opioid prescription at discharge, 65 911 received prescriptions before PDMP mandate implementation, and 27 849 received prescriptions after implementation. Most patients received general or obstetric/gynecologic surgery (48.6% and 30.1%, respectively), did not have diabetes (90.3%), and had never smoked (66.0%). Before the PDMP mandate was implemented, a decreasing pattern in opioid prescribing quantities was already occurring. During the quarter of implementation, total MMEs prescribed at discharge further decreased for all 3 surgical specialties (eg, medians for general surgery: β = −10.00 [95% CI, −19.52 to −0.48]; obstetric/gynecologic surgery: β = −18.65 [95% CI, −22.00 to −15.30]; and orthopedic surgery: β = −30.59 [95% CI, −40.19 to −21.00]) after adjusting for the preimplementation prescribing pattern. The total number of tablets prescribed also decreased across specialties (eg, medians for general surgery: β = −3.02 [95% CI, −3.47 to −2.57]; obstetric/gynecologic surgery: β = −4.86 [95% CI, −5.38 to −4.34]; and orthopedic surgery: β = −4.06 [95% CI, −5.07 to −3.04]) compared with the quarters before implementation. These reductions were not consistent across the most common surgical procedures. For cesarean delivery, the median number of tablets prescribed decreased during the quarter of implementation (β = −10.00; 95% CI, −10.10 to −9.90), but median MMEs did not (β = 0; 95% CI, −9.97 to 9.97), whereas decreases were observed in both median MMEs and number of tablets prescribed (MMEs: β = −33.33 [95% CI, −38.48 to −28.19]; tablets: β = −10.00 [95% CI, −11.17 to −8.82]) for laparoscopic cholecystectomy. For knee arthroscopy, no decreases were found in either median MMEs or number of tablets prescribed (MMEs: β = 10.00 [95% CI, −22.33 to 42.33; tablets: β = 0.83; 95% CI, −3.39 to 5.05). The proportion of prescriptions written for longer than 5 days also decreased significantly during the quarter of implementation across all 3 surgical specialties.

**Conclusions and Relevance:**

In this cross-sectional study, the implementation of mandatory PDMP consultation with a concurrent EHR-based alert was associated with an immediate decrease in opioid prescribing across the 3 surgical specialties. These findings might be explained by prescribers’ attempts to meet the mandate exemption and bypass PDMP consultation rather than the PDMP consultation itself. Although policies coupled with EHR alerts may be associated with changes in postoperative opioid prescribing behavior, they need to be well designed to optimize evidence-based opioid prescribing.

## Key Points

**Question:**

Was the implementation of mandatory consultation with a prescription drug monitoring program (PDMP) that integrates electronic health record (EHR) alerts associated with changes in the postoperative quantity of opioid medications prescribed at discharge after general, obstetric and gynecologic, and orthopedic surgery?

**Findings:**

In this cross-sectional study of 93 760 adult patients within a large health care system who received a postoperative prescription for an opioid medication, implementation of mandatory PDMP consultation with EHR-based alerts was associated with significant decreases in postoperative opioid prescription quantities during the quarter in which the mandate was implemented after accounting for preimplementation prescribing patterns.

**Meaning:**

This study found that implementation of legislation mandating PDMP consultation and the use of EHR alerts may be associated with changes in postoperative opioid prescribing behavior and decreases in the excess number of opioid tablets in circulation.

## Teaser

This cross-sectional study examines whether the implementation of mandated consultation with a prescription drug monitoring program was associated with changes in the quantity of postoperative opioid medications prescribed at discharge among adult patients in a health care system in California.

## Introduction

The overprescribing of opioid medications in the US has substantial implications for the current opioid crisis, with surgeons having the second highest rate of opioid prescribing.^1,2,3,4,5,6^ Studies have found that patients consume only a fraction of the opioid tablets prescribed to them, and more than two-thirds of postoperative tablets remain unused.^2,3,7,8,9,10^ Overprescribing of opioids has been associated with the development of chronic opioid use (with 6% of opioid-naive patients developing persistent opioid use) and the diversion of unused tablets, which can become a source for recreational opioid misuse.^11,12,13,14^ Because one-third of all prescriptions from surgeons are written for opioid medications, the development of measures to ensure appropriate and safe postoperative prescribing is paramount.^6^

As public attention surrounding the opioid crisis has increased, opioid prescribing has begun to decrease,^15^ and mechanisms to prevent misuse of prescription medications have been implemented. One mechanism to encourage safer opioid prescribing is the mandated use of prescription drug monitoring programs (PDMPs). These programs collect data on the prescribing and dispensing of controlled substances and have been used for more than a century after they were first introduced as law enforcement and regulatory tools.^16^ In response to the opioid crisis, several states have passed legislation mandating PDMP consultation to reduce prescription drug misuse and diversion. In July 2016, California passed legislation mandating the use of a PDMP entitled Controlled Utilization Review and Evaluation System (CURES).^17^ This mandate, which was implemented on October 2, 2018, requires prescribers to consult CURES before prescribing a controlled substance.

Although most studies examining the consequences of mandated PDMP consultation have reported an associated reduction in opioid prescribing, the data have been mixed.^18,19,20,21,22,23^ Few studies have focused on postoperative prescribing despite the fact that 80% of patients receive an opioid analgesic medication for pain management after surgery.^24,25^ Among studies that have examined postoperative prescribing, the focus has been on changes within 1 year,^26^ within a single specialty,^18,20,23^ or within pediatric populations.^22^ We examined 5 years of electronic health record (EHR) data from before and after the implementation of mandated CURES consultation prompted by an EHR-based alert. We investigated whether this mandatory PDMP consultation was associated with changes in the postoperative quantity of opioids prescribed at discharge after general, obstetric/gynecologic (obstetric/gynecologic), and orthopedic surgery among adult patients in a single health care system.

## Methods

This retrospective cross-sectional study was conducted at Sutter Health, a large integrated health care delivery system in northern California that includes 24 hospitals across 20 counties. The study was approved by the RAND Human Subjects Protection Committee; informed consent was not required because all data were deidentified. This study followed the Strengthening the Reporting of Observational Studies in Epidemiology (STROBE) reporting guideline for cross-sectional studies.

### Data Collection

We obtained retrospective EHR data from January 1, 2015, to February 1, 2020. The health care system examined uses an integrated EHR that is consistent across all hospitals. We included all adult patients who underwent general, obstetric/gynecologic, or orthopedic surgery; received an opioid prescription at discharge; and were discharged to home or self-care during this period. Patients were excluded if they had no record of an opioid prescription because we could not ensure that a prescription was missing because no prescription was written or a prescription was written but recorded in a different EHR system. We identified surgical specialties using EHR designations and selected the most commonly performed surgical procedure within each specialty. Patients were excluded if they underwent more than 1 operation during hospitalization (1.5% of the patients excluded), received an opioid prescription outside of the 4 most common types or quantities of opioid medications prescribed (hydrocodone-acetaminophen, 5-325 mg; hydrocodone-acetaminophen, 10-325 mg; oxycodone-acetaminophen, 5-325 mg; and oxycodone, 5 mg; 17.3% of the patients excluded), or received a total morphine milligram equivalent (MME) quantity higher than 900 (0.3% of the patients excluded). The number of patients excluded for each criterion and the rationale for exclusion criteria are available in eFigure 1 in the Supplement. We extracted both demographic and prescription data. The participating health care system has collected self-reported race and ethnicity data based on US census categories (American Indian or Alaska Native, Asian, Black or African American, Hispanic, Native Hawaiian or Pacific Islander, White, and other [with races and ethnicities in this category not specified]) since 2010.

### CURES Mandate Implementation

At the time the CURES mandate was implemented in October 2018, the health care system integrated a best-practice advisory into its EHR to prompt prescribers to consult CURES at the time of prescribing. The EHR-based best-practice advisories were used throughout the health care system examined. These alerts provide a hyperlink to the CURES online database, which prescribers access through a separate password-protected login process. Per California law, prescribers are exempt from this mandate during the postoperative period if they prescribe a 5-day or fewer supply of opioid medications, and they may select an exemption box on the best-practice advisory to bypass CURES consultation.^16^ Data were extracted from the 15 quarters before implementation (January 2015 to September 2018), the quarter during implementation (October to December 2018), and the 5 quarters after implementation (January 2019 to March 2020).

### Primary and Secondary Outcomes

The primary outcome was the total quantity of opioids (measured by means, medians, and 25th, 50th, and 75th percentiles of total MMEs and number of tablets) prescribed at discharge, regardless of whether the prescription was filled. The secondary outcome was the proportion of discharge opioid prescriptions with a duration longer than 5 days. These outcomes were analyzed by surgical specialty and most common surgical procedure performed within each specialty.

### Statistical Analysis

Patient characteristics before and after implementation of the CURES mandate were described using percentages, means with SDs, or medians with IQRs, as appropriate. The analysis of several descriptive features of discharge opioid quantities (means, medians, and 25th and 75th percentiles) was necessary to understand the full implications of the results because of the skewed distribution of MMEs and number of tablets prescribed (eFigure 2 in the Supplement). We were able to obtain prescription duration information for 78.4% of the analytic cohort; thus, the secondary outcome was analyzed only for patients who had these data available in the EHR.

An interrupted time series approach was used to adjust for preexisting patterns in outcomes before the CURES mandate was implemented.^27^ This approach compared observed prescribing after implementation of the CURES mandate with prescribing that would have been expected had the preimplementation prescribing patterns continued into the implementation period.^27^ Analyses were performed separately for each surgical specialty (general, obstetric/gynecologic, and orthopedic). We conducted analyses in which all surgical procedures for a given specialty were pooled, which provided greater statistical power and more generalizability than analyzing procedures separately. We also conducted analyses in which the most common procedure within each specialty (laparoscopic cholecystectomy for general surgery, cesarean delivery for obstetric/gynecologic surgery, and knee arthroscopy for orthopedic surgery) was restricted for better interpretability and contextualization. A linear regression analysis was used to assess the mean total MMEs and number of tablets prescribed, and SEs were clustered by prescriber to account for within-prescriber associations.^28^ A quantile regression analysis was used to analyze the 25th, 50th, and 75th percentiles of total MMEs and number of tablets prescribed at discharge. A logistic regression analysis was used to analyze the proportion of prescriptions with a duration of longer than 5 days. When pooling patients within a surgical specialty, a fixed effect for procedure type was included in the regression model to account for changes in the distribution of surgical procedures over time. All tests were 2-sided with statistical significance set at *P* < .05. Analyses were conducted using Stata software, version 16.0 (StataCorp LLC).

## Results

### Patient Characteristics

A total of 93 760 patients (mean [SD] age, 46.7 [17.6] years; 67.9% female; 32.1% male) met study criteria (65 911 from the preimplementation period and 27 849 from the postimplementation period). Patient demographic characteristics varied by surgical specialty (Table 1). Most patients received general or obstetric/gynecologic surgery (48.6% and 30.1%, respectively), did not have diabetes (90.3%), had never smoked (66.0%), and were categorized as having American Society of Anesthesiologists physical status of class 1 to 2 (12.2% with class 1 status and 57.0% with class 2) status, indicating they had normal health (class 1) or mild systemic disease (class 2). The clinical and demographic characteristics of patients before and after implementation of the CURES mandate, stratified by surgical specialty, are shown in eTable 1 in the Supplement.

**Table 1.** Patient Characteristics by Surgical Specialty

Characteristic	Surgical specialty, %			
	General	Obstetric and gynecologic	Orthopedic
Total participants, No.	45 597	28 207	19 956
Age, mean (SD), y	52.4 (17.3)	32.0 (6.1)	54.6 (16.7)
Sex^a^			
Female	56.9	>99.9	47.6
Male	43.1	<0.01	52.4
BMI, mean (SD)^b^	28.9 (6.2)	32.2 (6.1)	29.1 (5.9)
ASA status			
1-2	68.0	80.2	70.4
3-5	32.0	19.8	29.6
Presence of diabetes	12.3	3.6	12.4
Smoking status			
Never	60.9	78.9	59.4
Current	11.5	3.3	.8
Former	27.6	17.8	29.8
Race and ethnicity			
American Indian or Alaska Native	0.3	0.3	0.4
Asian	10.3	15.8	4.9
Black or African American	5.8	7.9	3.8
Hispanic	20.9	30.7	15.1
Native Hawaiian or Pacific Islander	0.5	0.8	0.3
White	56.2	36.6	69.0
Other^c^	6.0	7.9	6.5
Chronic opioid use^d^	6.9	1.3	10.2
Prescribing information			
No. of prescribers	994	633	535
Total MMEs prescribed in 24 h before discharge, median (IQR)	14.9 (7.5-26.4)	18.9 (10.0-32.5)	18.4 (10.0-36.0)
Total MMEs prescribed before CURES mandate			
Mean (SD)	198.2 (114.3)	189.0 (87.8)	360.9 (206.9)
Median (IQR)	150 (125-225)	150 (150-225)	300 (200-450)
Total MMEs prescribed after CURES mandate			
Mean (SD)	123.1 (72.1)	137.2 (62.7)	259.7 (138.0)
Median (IQR)	100 (75-150)	150 (100-150)	225 (150-315)
No. of tablets prescribed before CURES mandate			
Mean (SD)	32.6 (12.5)	31.9 (9.7)	48.0 (20.7)
Median (IQR)	30 (20-40)	30 (30-40)	40 (30-60)
No. of tablets prescribed after CURES mandate			
Mean (SD)	20.8 (9.6)	21.4 (8.3)	36.3 (15.7)
Median (IQR)	20 (15-30)	20 (15-30)	30 (28-42)

Abbreviations: ASA, American Society of Anesthesiologists; BMI, body mass index (calculated as weight in kilograms divided by height in meters squared); CURES, Controlled Utilization Review and Evaluation System; MME, morphine milligram equivalent.

^a^ Among patients who underwent obstetric or gynecologic surgery, 28 204 were female and 3 were male.

^b^ BMI outliers were removed by excluding values less than the 1st percentile and greater than the 99th percentile.

^c^ Races and ethnicities included in this category were not specified in the data set.

^d^ Chronic opioid use was defined as an outpatient prescription for an oral opioid recorded in the electronic health record within the 6 months before current admission, with a prescription duration longer than 90 days.

### Prescription Quantity

The quantity of opioids prescribed at discharge decreased after implementation of the CURES mandate compared with the years before implementation for all 3 specialties (Table 1). The mean (SD) total MMEs prescribed before vs after the CURES mandate was 198.2 (114.3) vs 123.1 (72.1) for general surgery, 189.0 (87.8) vs 137.2 (62.7) for obstetric/gynecologic surgery, and 360.9 (206.9) vs 259.7 (138.0) for orthopedic surgery, respectively; the median MMEs prescribed before vs after the CURES mandate was 150 (IQR, 125-225) vs 100 (IQR, 75-150) for general surgery, 150 (IQR, 150-225) vs 150 (IQR, 100-150) for obstetric/gynecologic surgery, and 300 (IQR, 200-450) vs 225 (150-315) for orthopedic surgery. The mean (SD) total number of tablets prescribed before vs after the CURES mandate was 32.6 (12.5) vs 20.8 (9.6) for general surgery, 31.9 (9.7) vs 21.4 (8.3) for obstetric/gynecologic surgery, and 48.0 (20.7) vs 36.3 (15.7) for orthopedic surgery, respectively; the median total number of tablets prescribed before vs after the CURES mandate was 30 (IQR, 20-40) vs 20 (IQR, 15-30) for general surgery, 30 (IQR, 30-40) vs 20 (IQR, 15-30) for obstetric/gynecologic surgery, and 40 (IQR, 30-60) vs 30 (IQR, 28-42) for orthopedic surgery.

Before the CURES mandate, a significantly decreasing pattern was already occurring in all opioid quantities prescribed at discharge across all specialties (eg, general surgery: median MMEs, β = −6.55 [95% CI, −7.32 to −5.79; *P* < .001]; median number of tablets, β = −0.91 [95% CI, −0.94 to −0.88; *P* < .001]) (Table 2). Results from the regression analysis revealed that at the time the CURES mandate was implemented, there was an additional decrease in all measures of opioid prescribing for all 3 specialties (eg, general surgery: median MMEs, β = −10.00 [95% CI, −19.52 to −0.48; *P* = .04]; median number of tablets, β = −3.02 [95% CI, −3.47 to −2.57; *P* < .001]). After adjusting for preimplementation patterns, for general surgery, the mean total MMEs prescribed at discharge decreased by 5.3% (β = −9.30; 95% CI, −18.31 to −0.29; *P* = .04), and the median MMEs decreased by 7.0% (β = −10.00; 95% CI, −19.52 to −0.48; *P* = .04) during the quarter of implementation but remained at the same level thereafter with no further decreases that significantly deviated from the preimplementation pattern. The mean total number of tablets prescribed decreased by 10.5% (β = −2.33; 95% CI, −3.85 to −0.81), and the median number of tablets decreased by 12.5% (β = −3.02; 95% CI, −3.47 to −2.57) during the quarter of implementation and remained lower at 4 quarters after the CURES mandate (median, β = −2.37 [95% CI, −2.86 to −1.89; *P* < .001]; mean, β = −2.43 [95% CI, −4.82 to −0.04; *P* = .046]) (Table 2).

**Table 2.** Interrupted Time Series Regression Analysis of Opioid Quantities Prescribed Before and After Implementation of the CURES Mandate

Surgical specialty	MMEs prescribed, β coefficient (95% CI)		No. of tablets prescribed, β coefficient (95% CI)	
	Mean^a^	Median^b^	Mean^a^	Median^b^
General surgery				
Quarters before implementation^c^	−7.61 (−9.41 to −5.80)^d^	−6.55 (−7.32 to −5.79)^d^	−0.92 (−1.14 to −0.70)^d^	−0.91 (−0.94 to −0.88)^d^
Quarter of implementation^e^	−9.30 (−18.31 to −0.29)^f^	−10.00 (−19.52 to −0.48)^f^	−3.07 (−4.41 to −1.73)^d^	−3.02 (−3.47 to −2.57)^d^
Quarters after implementation^g^				
1	−3.50 (−14.00 to 7.00)	−4.60 (−14.36 to 5.16)	−2.33 (−3.85 to −0.81)^h^	−2.26 (−2.72 to −1.80)^d^
2	−4.78 (−16.77 to 7.21)	−6.69 (−12.54 to 8.47)	−3.18 (−4.92 to −1.44)^d^	−3.09 (−3.55 to −2.64)^d^
3	1.51 (−14.54 to 17.56)	−2.04 (−7.66 to 14.07)	−2.75 (−4.97 to −0.52)^i^	−2.69 (−3.16 to −2.21)^d^
4	7.75 (−9.39 to 24.90)	3.21 (−7.66 to 14.07)	−2.43 (−4.82 to −0.04)^j^	−2.37 (−2.86 to −1.89)^d^
5	8.71 (−9.57 to 26.98)	4.21 (−10.43 to 18.84)	−2.26 (−4.78 to 0.26)	−2.20 (−2.88 to −1.51)^d^
Obstetric and gynecologic surgery				
Quarters before implementation^c^	−3.77 (−4.85 to −2.69)^d^	−3.25 (−3.50 to −2.99)^d^	−0.65 (−0.77 to −0.53)^d^	−0.63 (−0.66 to −0.60)^d^
Quarter of implementation^e^	−21.64 (−32.22 to −11.06)^d^	−18.65 (−22.00 to −15.30)^d^	−4.63 (−5.96 to −3.30)^d^	−4.86 (−5.38 to −4.34)^d^
Quarters after implementation^g^				
1	−22.80 (−33.85 to −11.74)^d^	−20.10 (−23.58 to −16.63)^d^	−5.07 (−6.41 to −3.73)^d^	−5.27 (−5.79 to −4.76)^d^
2	−22.13 (−32.66 to −11.59)^d^	−19.75 (−23.44 to −16.05)^d^	−5.14 (−6.48 to −3.80)^d^	−5.34 (−5.88 to −4.80)^d^
3	−19.01 (−30.09 to −7.92)^k^	−17.44 (−21.22 to −13.66)^d^	−4.88 (−6.34 to −3.42)^d^	−5.07 (−5.59 to −4.54)^d^
4	−10.01 (−23.57 to 3.54)	−10.57 (−14.81 to −6.34)^d^	−3.87 (−5.71 to −2.02)^d^	−4.17 (−4.76 to −3.59)^d^
5	−4.95 (−20.98 to 11.07)	−6.74 (−13.16 to −0.33)^f^	−3.36 (−5.53 to −1.20)^l^	−3.68 (−4.58 to −2.78)^d^
Orthopedic surgery				
Quarters before implementation^c^	−9.91 (−12.67 to −7.16)^d^	−9.20 (−9.98 to −8.42)^d^	−1.08 (−1.37 to −0.79)^d^	−1.07 (−1.14 to −1.00)^d^
Quarter of implementation^e^	−31.57 (−53.91 to −9.22)^m^	−30.59 (−40.19 to −21.00)^d^	−4.07 (−6.51 to −1.64)^k^	−4.06 (−5.07 to −3.04)^d^
Quarters after implementation^g^				
1	−35.71 (−59.72 to −11.70)^n^	−35.49 (−45.47 to −25.51)^d^	−4.11 (−6.93 to −1.29)^n^	−4.11 (−5.16 to −3.05)^d^
2	−42.43 (−67.02 to −17.85)^k^	−41.16 (−50.94 to −31.37)^d^	−4.91 (−8.07 to −1.74)^l^	−4.87 (−5.90 to −3.84)^d^
3	−42.68 (−67.13 to −18.22)^k^	−42.19 (−52.67 to −31.71)^d^	−5.54 (−8.41 to −2.66)^d^	−5.50 (−6.57 to −4.42)^d^
4	−28.15 (−53.94 to −2.37)^o^	−28.06 (−38.66 to −17.45)^k^	−4.10 (−7.29 to −0.91)^i^	−4.06 (−5.13 to −2.99)^d^
5	−8.72 (−38.81 to 21.37)	−9.95 (−24.72 to 4.83)	−1.84 (−5.28 to 1.59)	−1.81 (−3.32 to −0.31)^i^

Abbreviations: CURES, Controlled Utilization Review and Evaluation System; MME, morphine milligram equivalent.

^a^ Interrupted time series analysis was performed using linear regression of mean MME, clustering SEs by prescriber and using surgical specialty as a fixed effect.

^b^ Interrupted time series analysis was performed using quantile regression of MME (with medians shown in table) using surgical specialty as a fixed effect.

^c^ Includes the 15 quarters from January 2015 to September 2018.

^d^ *P* < .001.

^e^ Includes the quarter from October to December 2018.

^f^ *P* = .04.

^g^ Includes the 5 quarters from January 2019 to March 2020 (quarter 1, January to March 2019; quarter 2, April to June 2019; quarter 3, July to September 2019; quarter 4, October to December 2019; and quarter 5, January to March 2020).

^h^ *P* = .003.

^i^ *P* = .02.

^j^ *P* = .046.

^k^ *P* = .001.

^l^ *P* = .002.

^m^ *P* = .006.

^n^ *P* = .004.

^o^ *P* = .03.

When pooling all obstetric/gynecologic surgery prescriptions, the adjusted mean total MMEs prescribed at discharge decreased by 12.2% (β = −21.64; 95% CI, −32.22 to −11.06; *P* < .001), and the median MMEs decreased by 12.7% (β = −18.65; 95% CI, −22.00 to −15.30; *P* < .001) during the quarter of implementation and remained lower at 2 quarters after implementation (median, β = −19.76 [95% CI, −23.44 to −16.05; *P* < .001]; mean, β = −22.13 [95% CI, −32.66 to −11.59; *P* < .001]; difference in mean total MMEs was not significant at 4 and 5 quarters after implementation). The mean total number of tablets prescribed decreased by 15.7% (β = −4.63; 95% CI, −5.96 to −3.30), and the median number of tablets decreased by 14.0% (β = −4.86; 95% CI, −5.38 to −4.34) during the quarter of implementation and remained lower at 5 quarters after the CURES mandate (median, β = −3.58 [95% CI, −4.58 to −2.78; *P* < .001]; mean, β = −3.36 [95% CI, −5.53 to −1.20; *P* = .002]) (Table 2).

For orthopedic surgery, the adjusted mean total MMEs prescribed at discharge decreased by 9.6% (β = −31.57; 95% CI, −53.91 to −9.22), and the median MMEs decreased by 11.3% (β = −30.59; 95% CI, −40.19 to −21.00) during the quarter of implementation and remained lower at 4 quarters after implementation (median, β = −28.05 [95% CI, −38.66 to −17.45; *P* < .001]; mean, β = −28.15 [95% CI, −53.94 to −2.37; *P* = .03]; difference in mean total MMEs was not significant at 5 quarters after implementation). The mean total number of tablets prescribed decreased by 9.2% (β = −4.07; 95% CI, −6.51 to −1.64), and the median total number of tablets decreased by 10.4% (β = −4.06; 95% CI, −5.07 to −3.04) and remained lower at 4 quarters after implementation of the CURES mandate (median, β = −4.06 [95% CI, −5.13 to −2.99; *P* < .001]; mean, β = −4.10 [95% CI, −7.29 to −0.91; *P* = .01]) (Table 2). Analysis of the 25th and 75th percentiles of total MMEs and number of tablets prescribed revealed similar results (eTable 2 in the Supplement).

Based on the number of procedures performed across the 3 specialties and the effect sizes (shown in Table 2), we estimated that the CURES mandate was associated with more than 100 000 fewer opioid tablets in circulation after 5 quarters of implementation in this health care system alone (eTable 4 in the Supplement).

### Laparoscopic Cholecystectomy

**Figure 1.**
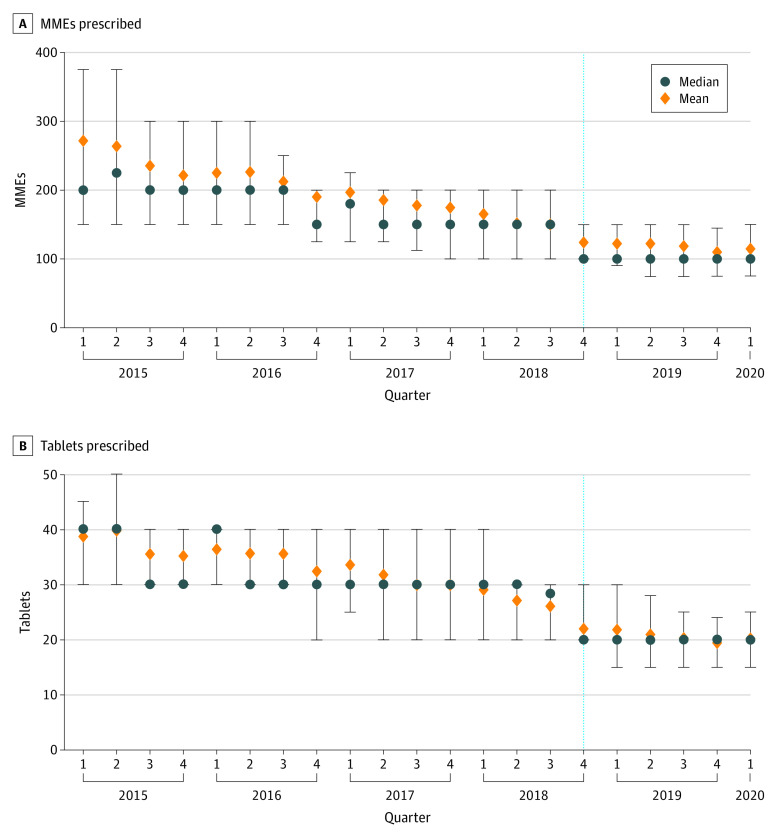
Opioid Prescribing for Laparoscopic Cholecystectomy Before and After Implementation of CURES Mandate The dashed line represents the quarter in which the CURES mandate was implemented. Error bars indicate IQR. CURES indicates Controlled Utilization Review and Evaluation System and MME, morphine milligram equivalent.

Laparoscopic cholecystectomy was the most common general surgical procedure, with 6922 operations performed in the preimplementation period and 2613 in the postimplementation period. The quantity of opioids prescribed at discharge after laparoscopic cholecystectomy followed a decreasing pattern in mean total MMEs prescribed per quarter (Figure 1). In the quarter during which CURES was mandated, the median total MMEs prescribed decreased by one-third, from 150 in the 6 quarters before implementation to 100 during the quarter of implementation (β = −33.33; 95% CI, −38.48 to −28.19; *P* < .001). The median total number of tablets prescribed decreased from 25 tablets in the quarter before implementation to 20 tablets during the quarter of implementation (β = −10.00; 95% CI, −11.17 to −8.82; *P* < .001). The median total MMEs and number of tablets prescribed at discharge remained the same in the quarters thereafter, without any further decreases (Figure 1; eTable 3 in the Supplement). Overall, opioid prescribing for laparoscopic cholecystectomy followed the same pattern as the mean for all general surgery prescriptions.

### Cesarean Delivery

**Figure 2.**
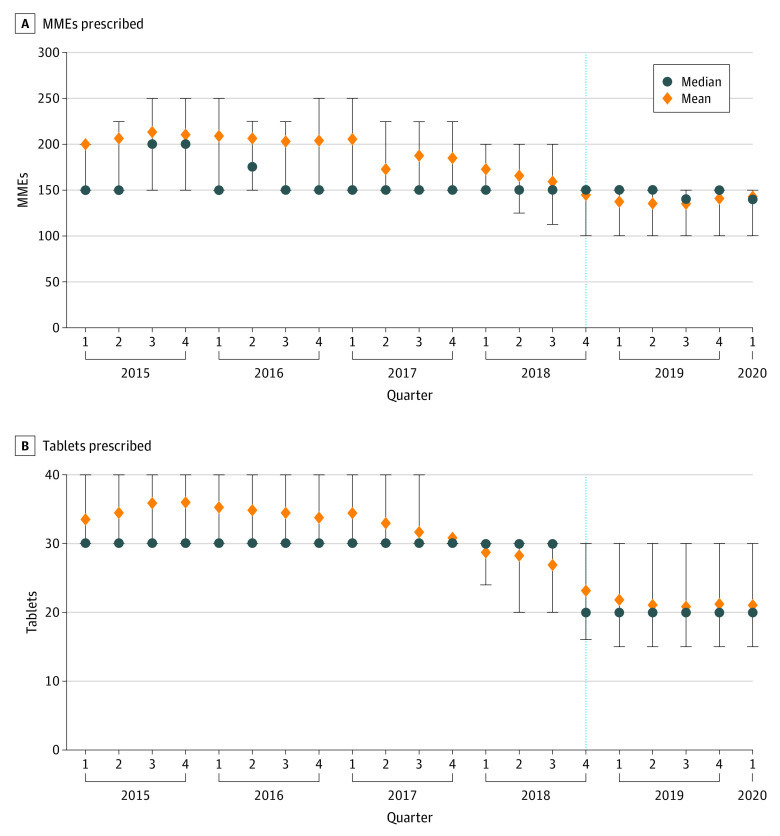
Opioid Prescribing for Cesarean Delivery Before and After Implementation of CURES Mandate The dashed line represents the quarter in which the CURES mandate was implemented. Error bars indicate IQR. CURES indicates Controlled Utilization Review and Evaluation System and MME, morphine milligram equivalent.

Cesarean delivery was the most common obstetric/gynecologic surgical procedure, with 18 946 operations performed in the preimplementation period and 6830 in the postimplementation period. Although mean total MMEs prescribed at discharge had a slightly decreasing pattern before implementation of the CURES mandate (β  **=**  −3.89; 95% CI, −5.05 to −2.73), there was no decrease in median total MMEs during the quarter of implementation (β  **=**  0; 95% CI, −9.97 to 9.97; *P* > .99) (Figure 2A; eTable 3 in the Supplement). Median total MMEs were the same as the postimplementation median for all obstetric/gynecologic prescriptions (preimplementation: 150 MMEs [IQR, 150-225 MMEs]; postimplementation: 150 MMEs [IQR, 100-150 MMEs]), and mean (SD) total MMEs were higher than the postimplementation mean for all obstetric/gynecologic prescriptions (preimplementation: 189.0 [87.8] MMEs; postimplementation: 137.2 [62.7] MMEs). Although analysis of the pooled obstetric/gynecologic prescriptions revealed a decrease in all 4 opioid prescribing outcomes during the quarter of CURES mandate implementation, the median number of tablets prescribed for cesarean delivery decreased by one-third (from 30 tablets to 20 tablets; β = −10.00; 95% CI, −10.10 to −9.90; *P* < .001) without a concurrent decrease in the median total MMEs prescribed (Figure 2B; eTable 3 in the Supplement).

To better understand these results, we performed an analysis of the type of opioid medication prescribed. In the quarter during which the CURES mandate was implemented, prescribers switched from hydrocodone-acetaminophen to oxycodone, with the proportion of prescriptions for oxycodone, 5 mg, increasing by the same amount that the proportion of prescriptions for hydrocodone-acetaminophen and oxycodone-acetaminophen decreased (eFigure 3 in the Supplement).

### Knee Arthroscopy

**Figure 3.**
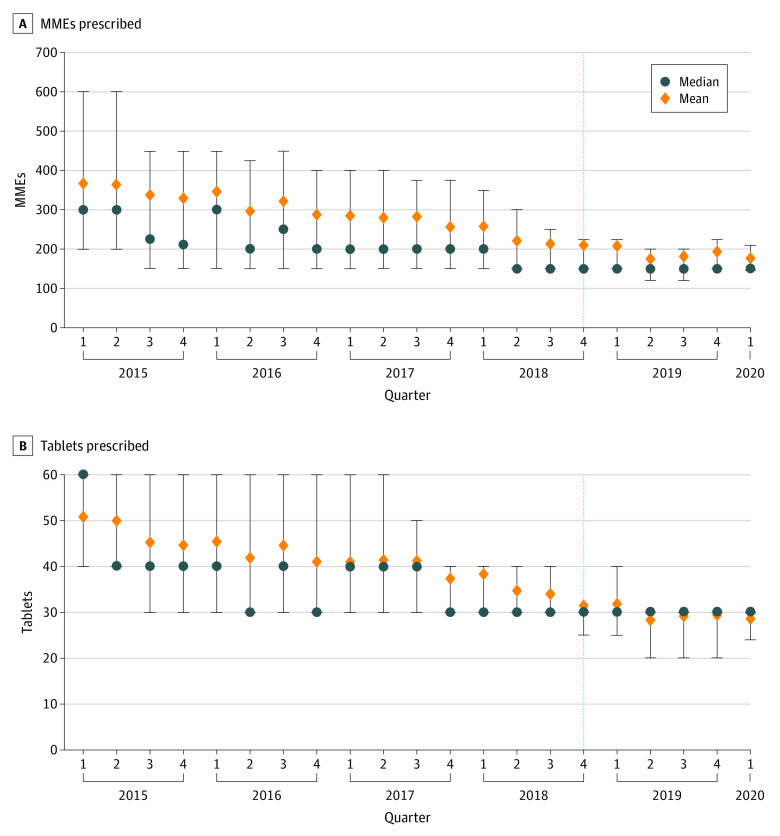
Opioid Prescribing for Knee Arthroscopy Before and After Implementation of CURES Mandate The dashed line represents the quarter in which the CURES mandate was implemented. Error bars indicate IQR. CURES indicates Controlled Utilization Review and Evaluation System and MME, morphine milligram equivalent.

Knee arthroscopy was the most common orthopedic surgical procedure, with 2090 operations performed in the preimplementation period and 828 in the postimplementation period. During the quarter of implementation, the median total MMEs (β = 10.00; 95% CI, −22.33 to 42.33; *P* = .54) and number of tablets (β = 0.83; 95% CI, −3.39 to 5.05; *P* = .70) prescribed at discharge did not decrease (Figure 3; eTable 3 in the Supplement). At the time of implementation, the mean and median total MMEs and number of tablets prescribed were already at or lower than the postimplementation means and medians (mean [SD], 259.7 [138.0] MMEs; median, 225 MMEs [IQR, 150-315 MMEs]) for pooled orthopedic prescriptions. Unlike the mean across all orthopedic surgery prescriptions, opioid prescribing for knee arthroscopy did not significantly change during the quarter in which the CURES mandate was implemented for any of the 4 outcomes.

### Duration of Prescriptions

The proportion of discharge opioid prescriptions with a duration longer than 5 days was lower in the years after vs before implementation of the CURES mandate for all 3 specialties. Before the CURES mandate, the proportion of discharge opioid prescriptions with a duration longer than 5 days was already significantly decreasing (eg, general surgery: β = −0.10; 95% CI, −0.11 to 0.10; *P* < .001) (eFigure 4 and eTable 5 in the Supplement). During the quarter in which the CURES mandate was implemented, there was an additional decrease in this proportion for all 3 specialties (eg, general surgery: β = −0.53; 95% CI, −0.65 to −0.40; *P* < .001), with a 10.2% reduction (β = −0.10; 95% CI, −0.12 to −0.08; *P* < .001) for general surgery, a 9.7% reduction (β = −0.97; 95% CI, −0.13 to −0.07; *P* < .001) for obstetric/gynecologic surgery, and a 6.9% reduction (β = −0.69; 95% CI, −0.10 to −0.4; *P* < .001) for orthopedic surgery (eTable 5 in the Supplement).

## Discussion

In this cross-sectional study, across 3 surgical specialties, postoperative opioid prescribing at discharge decreased after implementation of the CURES mandate and the concurrently integrated EHR alert, which was a statistically significant and clinically meaningful finding. Although prescription quantities were already decreasing before the mandate, the decrease that occurred after the mandate was a statistically significant deviation from the preimplementation pattern. We estimated that the CURES mandate was associated with more than 100 000 fewer opioid tablets in circulation after 5 quarters of CURES mandate implementation in this health care system alone (eTable 4 in the Supplement). We also found that the proportion of opioid prescriptions written at discharge for longer than 5 days decreased at the time of implementation. If these estimates are consistent across other hospital systems and specialties, the findings could have substantial implications for reducing the number of opioid tablets statewide.

The implementation of an EHR alert to prompt adherence to the CURES mandate introduced a disruption to physician workflow at the health care system examined. At the time of prescribing, the EHR alert requires that physicians open the PDMP in a separate browser, enter their CURES login information, and re-enter their patient’s information to view previous prescriptions. However, postoperative prescribers who write prescriptions for no more than a 5-day supply can proceed without having to consult CURES. At a schedule of 1 dose every 6 hours, a 5-day supply is 20 tablets. The mandate exemption and opportunity to circumvent the CURES consultation might explain why the median number of tablets prescribed for the most common general and obstetric/gynecologic surgical procedures decreased to exactly 20 tablets when the CURES mandate was implemented, allowing prescribers to bypass the extra consultation step. Alert fatigue is a well-researched issue, with reports finding that physicians override or ignore 49% to 96% of all alerts.^29,30^ Strategies (in this case, changing a prescription) to avoid such alerts are common in the medical setting.^31^

Although this study cannot separate the impact of the law from the best-practice advisory, the findings suggest that reduced prescribing may be, in part, a reaction to the implementation of the EHR alerts rather than the CURES consultation requirement itself. This interpretation is strengthened by the significant decrease in prescriptions written for longer than 5 days at the time of CURES mandate implementation. The 5-day prescribing exemption may also explain the differences in effect sizes between surgical specialties. General surgery had lower prescribing quantities before the CURES mandate; thus, the change in prescribing needed to reach the exemption level was smaller, with an estimated required decrease in median total MMEs and number of tablets of only 5% and 3 tablets, respectively. However, obstetric/gynecologic and orthopedic operations started with higher mean total MMEs and number of tablets prescribed, resulting in a larger decrease in prescribing quantities (a reduction of more than 10% in mean total MMEs and 4-5 median total tablets prescribed) to meet the exemption and bypass the consultation.

Our results suggest that specifics matter. Although several large academic medical centers have independently formulated guidelines on postoperative prescribing based on opioid quantity and type rather than prescription duration,^32,33,34^ the CURES mandatory consultation exemption is based on duration of prescription, not quantity or type of opioid prescribed.^16^ This discrepancy between a legislative exemption based on duration and guidelines based on quantity was most clearly observed in our results for cesarean delivery. Although the number of tablets prescribed decreased, which likely occurred in an effort to meet the postoperative exemption (20 tablets), prescribers switched from a less potent (hydrocodone) to a more potent (oxycodone) opioid medication, producing no change in total MMEs prescribed. The median total MMEs after implementation for both cesarean delivery and laparoscopic cholecystectomy remained at or above the maximum guidelines for these procedures after implementation of the CURES mandate, suggesting that one-half of all prescribers continued to prescribe higher than the maximum recommended opioid quantity. If the exemption and concurrently implemented EHR alert had explicitly used a maximum total MME dosage (based on evidence-based guidelines) rather than prescription duration, it is possible that the consequences of the CURES mandate would have been more pronounced.

### Strengths and Limitations

This study has strengths. Previous studies in this field examined preimplementation data for only 10 months or less of prescribing.^18,20,22,26^ Furthermore, 3 studies included only 1 surgical specialty,^18,20,23^ and 1 study examined a pediatric population.^22^ Strengths of our study compared with previous work include a large EHR-based data set from 24 hospitals and numerous surgical specialties during a period of 5 years. In particular, the study database comprised 15 months of preimplementation data, allowing for a more robust estimate of opioid prescribing patterns before the CURES mandate.

The study also has limitations. First, the implementation of the CURES mandate did not occur in isolation of other efforts to limit postoperative opioid prescribing. The potential consequences of these other efforts may be most clearly observed in the findings for knee arthroscopy, in which median total MMEs had already decreased to lower than the recommended guidelines in the preceding quarters, possibly in response to local opioid prescribing initiatives, and did not reflect a further decrease in prescribing at the time of CURES mandate implementation. Similar initiatives may have encouraged prescribers to switch from hydrocodone-acetaminophen to oxycodone after cesarean delivery in the quarter of interest. Our study cannot account for confounding policies or implementation efforts occurring in the health care system examined. We are not aware of any concurrent policies; however, if other opioid prescribing policies coincided with CURES mandate implementation, our results could be overstated.

Second, we studied a single health care system in northern California, which limits the generalizability of our findings. Mandates for PDMPs may motivate prescribers differently, both within California and across states. Third, we were not able to include patients for whom no opioid was prescribed. Thus, if the CURES mandate produced fewer opioid prescriptions, our results would be underestimated. Fourth, the interrupted time series analysis assumes that pre-CURES prescribing patterns continued in the absence of CURES mandate implementation. As time progresses farther from initial implementation, such extrapolation becomes less assured. Fifth, we do not have data regarding whether the decrease in opioid prescription quantities may have negative consequences for patient-associated outcomes, such as patient satisfaction, medication refills, or rehospitalizations because of pain. Moreover, our outcomes captured prescribing behavior and did not measure potential downstream consequences, such as whether a medication was dispensed or filled, whether opioid-associated overdoses occurred, or whether new diagnoses of opioid use disorder were made. Thus, it is not clear whether the implementation of the CURES mandate was associated with improvements in clinical and public health outcomes.

## Conclusions

The findings of this study suggest that a PDMP consultation mandate coupled with an EHR-based alert may successfully and efficiently change opioid prescribing behavior at discharge. However, the data suggest that the implementation of EHR alerts, along with the postoperative exemption to the CURES mandate (intended to reduce the burden of consulting CURES in certain circumstances), may have had the unintended consequences of weakening the benefits of the law and the changes to prescribing patterns as recommended by guidelines. This possibility highlights the importance of using implementation strategies to support the underlying goal of legislative policy. Implementing well-designed policies in conjunction with educational efforts may be the most successful means of reducing excess opioid prescribing.

## Notes

Supplement. **eTable 1.** Patient Characteristics Before and After CURES Mandate Implementation **eTable 2.** 25th and 75th Percentile Interrupted Time Series Regression Analysis Results by Surgical Specialty **eTable 3.** Interrupted Time Series Regression Analysis Results By Type of Surgery **eTable 4.** Inputs for Calculation of Tablet Reduction **eTable 5.** Interrupted Time Series Regression Analysis for 5-Day Duration **eFigure 1.** Patient Flowchart and Exclusion Criteria Rationale **eFigure 2.** Distribution of Morphine Milligram Equivalents and Number of Tablets **eFigure 3.** Change in Opioid Type Prescribed for Cesarean Delivery at Time of CURES Mandate Implementation **eFigure 4.** Patterns in Prescriptions With a Greater Than 5-Day Duration
