# Supplementary material for: Assessment of Postoperative Opioid Prescriptions Before and After Implementation of a Mandatory Prescription Drug Monitoring Program
Source: JAMA Health Forum. 2021 Oct 1;2(10):e212924. doi: 10.1001/jamahealthforum.2021.2924 (PMC8725834; doi:10.1001/jamahealthforum.2021.2924)
Supplement: Supplement. — eTable 1. Patient Characteristics Before and After CURES Mandate Implementation eTable 2. 25th and 75th Percentile Interrupted Time Series Regression Analysis Results by Surgical Specialty eTable 3. Interrupted Time Series Regression Analysis Results By Type of Surgery eTable 4. Inputs for Calculation of Tablet Reduction eTable 5. Interrupted Time Series Regression Analysis for 5-Day Duration eFigure 1. Patient Flowchart and Exclusion Criteria Rationale eFigure 2. Distribution of Morphine Milligram Equivalents and Number of Tablets eFigure 3. Change in Opioid Type Prescribed for Cesarean Delivery at Time of CURES Mandate Implementation eFigure 4. Patterns in Prescriptions With a Greater Than 5-Day Duration [file jamahealthforum-e212924-s001.pdf]

Supplementary Online Content

Shenoy R, Wagner Z, Kirkegaard A, et al. Assessment of postoperative opioid prescriptions before and after implementation of a mandatory prescription drug monitoring program. *JAMA Health Forum*. 2021;2(10):e212924. doi:10.1001/jamahealthforum.2021.2924

eTable 1. Patient Characteristics Before and After CURES Mandate Implementation

eTable 2. 25th and 75th Percentile Interrupted Time Series Regression Analysis Results by Surgical Specialty

eTable 3. Interrupted Time Series Regression Analysis Results By Type of Surgery

eTable 4. Inputs for Calculation of Tablet Reduction

eTable 5. Interrupted Time Series Regression Analysis for 5-Day Duration

eFigure 1. Patient Flowchart and Exclusion Criteria Rationale

eFigure 2. Distribution of Morphine Milligram Equivalents and Number of Tablets

eFigure 3. Change in Opioid Type Prescribed for Cesarean Delivery at Time of CURES Mandate Implementation

eFigure 4. Patterns in Prescriptions With a Greater Than 5-Day Duration

This supplementary material has been provided by the authors to give readers additional information about their work.

© 2021 Shenoy R et al. *JAMA Health Forum*.

eTable 1. Patient Characteristics Before and After CURES Mandate Implementation

**A. General Surgery**		
	Pre	Post
Number of Patients	31,803	13,794
Age (years), mean ± SD	52.9 ± 17.2	52.2 ± 17.3
Sex (female)	57.0%	56.7%
BMI[a], mean ± SD	28.8 ± 6.2	29.0 ± 6.3
ASA Status		
1-2	68.2%	67.7%
3-5	31.8%	32.3%
Diabetes (yes)	12.4%	12.1%
Smoking		
Never Smoker	60.5%	61.8%
Current Smoker	11.8%	10.6%
Former Smoker	27.6%	27.6%
Race/Ethnicity		
White	56.9%	54.5%
Hispanic	20.9%	20.8%
Black or African-American	5.5%	6.3%
Asian	10.1%	10.7%
Native Hawaiian/Pacific Islander	0.5%	0.6%
American Indian/Alaska Native	0.3%	0.4%
Other	5.7%	6.8%
Chronic Opioid User (Yes), %	7.7%	5.2%
Total MME Prescribed in 24 Hours Prior to Discharge, median [IQR]	15 [7.5, 27.5]	12.7 [6.4, 22.5]
**B. Obstetric/Gynecologic Surgery**		
	Pre	Post
Number of Patients	20,899	7,308
Age (years), mean ± SD	32.0 ± 6.1	32.2 ± 5.9
Sex (female)	>99.9%	>99.9%
BMI, mean ± SD	32.2 ± 6.1	32.5 ± 6.1
ASA Status		
1-2	80.7%	79.0%
3-5	19.3%	21.0%
Diabetes (yes)	3.7%	3.3%
Smoking		
Never Smoker	78.7%	79.5%

© 2021 Shenoy R et al. *JAMA Health Forum*.

Current Smoker	3.3%	3.2%
Former Smoker	17.9%	17.4%
Race/Ethnicity		
White	37.2%	34.9%
Hispanic	30.6%	31.0%
Black or African-American	7.7%	8.6%
Asian	16.0%	15.4%
Native Hawaiian/Pacific Islander	0.8%	0.8%
American Indian/Alaska Native	0.3%	0.4%
Other	7.5%	8.9%
Chronic Opioid User (Yes), %	1.5%	0.7%
Total MME Prescribed in 24 Hours Prior to Discharge, median [IQR]	19.0 [10, 35]	18.0 [10, 30]
**C. Orthopedic Surgery**		
	Pre	Post
Number of Patients	13,209	6,757
Age (years), mean ± SD	53.7 ± 16.7	56.2 ± 16.6
Sex (female)	47.4%	48.1%
BMI, mean ± SD	29.2 ± 6.0	29.1 ± 5.8
ASA Status		
1-2	70.5%	70.2%
3-5	29.5%	29.8%
Diabetes (yes)	12.4%	12.5%
Smoking		
Never Smoker	59.2%	59.8%
Current Smoker	11.3%	9.8%
Former Smoker	29.4%	30.4%
Race/Ethnicity		
White	68.6%	69.8%
Hispanic	15.6%	14.1%
Black or African-American	3.7%	3.9%
Asian	4.8%	5.0%
Native Hawaiian/Pacific Islander	0.4%	0.3%
American Indian/Alaska Native	0.4%	0.3%
Other	6.5%	6.6%
Chronic Opioid User (Yes), %	11.3%	8.0%
Total MME Prescribed in 24 Hours Prior to Discharge, median [IQR]	18.6 [10, 36.4]	18.2 [10, 34.5]

[a] BMI Outliers removed by excluding values less than 1st percentile and greater than 99th percentile
Abbreviations: SD, standard deviation; ns, not significant; BMI, body mass index; ASA, American Society of Anesthesiologists

eTable 2. 25th and 75th Percentile Interrupted Time Series Regression Analysis Results by Surgical Specialty[a]

A. General Surgery				
	25th Percentile MME	75th Percentile MME	25th Percentile Tablet Number	75th Percentile Tablet Number
	Coefficient (95% Confidence Interval)	*Coefficient (95% Confidence Interval)*	*Coefficient (95% Confidence Interval)*	*Coefficient (95% Confidence Interval)*
Pre-Implementation Trend	-4.58[d] (-4.89, -4.27)	-9.64[d] (-11.72, -7.57)	-0.83[d] (-0.87, -0.80)	-1.00[d] (-1.04, -0.95)
Quarter of Implementation	-11.31[d] (-15.09, -7.54)	-7.94 (-33.82, 17.95)	-2.50[d] (-3.06, -1.95)	-3.57[d] (-4.21, -2.94)
1 Quarter After Implementation	-6.67[c] (-10.54, -2.79)	-1.36 (-27.89, 25.17)	-1.56[d] (-2.12, -1.00)	-3.01[d] (-3.65, -2.36)
2 Quarters After Implementation	-10.28[d] (-14.28, -6.27)	-1.06 (-28.50, 26.37)	-2.16[d] (-2.72, -1.60)	-4.08[d] (-4.73, -3.44)
3 Quarters After Implementation	-8.69[d] (-12.86, -4.52)	8.34 (-20.15, 36.94)	-2.10[d] (-2.69, -1.52)	-3.31[d] (-3.99, -2.64)
4 Quarters After Implementation	-5.32[b] (-9.64, -1.00)	16.58 (-12.95, 46.11)	-1.79[d] (-2.39, -1.19)	-3.00[d] (-3.68, -2.31)
5 Quarters After Implementation	-4.22 (-10.03, 1.59)	17.43 (-22.36, 57.22)	-1.61[d] (-2.46, -0.77)	-2.82[d] (-3.79, -1.85)
**B. Obstetric/Gynecologic Surgery**				
	25th Percentile MME	75th Percentile MME	25th Percentile Tablet Number	75th Percentile Tablet Number
	Coefficient (95% Confidence Interval)	*Coefficient (95% Confidence Interval)*	*Coefficient (95% Confidence Interval)*	*Coefficient (95% Confidence Interval)*
Pre-Implementation Trend	-2.69[d] (-2.96, -2.41)	-4.39[d] (-4.79, -3.99)	-0.54[d] (-0.58, -0.51)	-0.79[d] (-0.84, -0.74)
Quarter of Implementation	-15.45[d] (-19.07, -11.83)	-25.16[d] (-30.35, -19.96)	-5.61[d] (-6.18, -5.03)	-3.45[d] (-4.27, -2.62)
1 Quarter After Implementation	-17.23[d] (-20.99, -13.47)	-25.96[d] (-31.34, -20.57)	-5.92[d] (-6.49, -5.36)	-4.04[d] (-4.86, -3.22)
2 Quarters After Implementation	-17.21[d] (-21.21, -13.21)	-24.92[d] (-30.64, -19.19)	-6.00[d] (-6.60, -5.41)	-4.09[d] (-4.95, -3.24)
3 Quarters After Implementation	-15.76[d] (-19.85, -11.68)	-20.85[d] (-26.70, -14.99)	-5.68[d] (-6.25, -5.10)	-3.91[d] (-4.74, -3.08)
4 Quarters After Implementation	-11.17[d] (-15.75, -6.59)	-9.36[c] (-15.92, -2.80)	-5.18[d] (-5.83, -4.54)	-2.27[d] (-3.20, -1.33)

© 2021 Shenoy R et al. *JAMA Health Forum*.

5 Quarters After Implementation	-8.65[c] (-15.59, -1.71)	-2.86 (-12.80, 7.08)	-4.72[d] (-5.71, -3.73)	-1.72[b] (-3.14, -0.29)
**C. Orthopedic Surgery**				
	25th Percentile MME	75th Percentile MME	25th Percentile Tablet Number	75th Percentile Tablet Number
	Coefficient (95% Confidence Interval)	*Coefficient (95% Confidence Interval)*	*Coefficient (95% Confidence Interval)*	*Coefficient (95% Confidence Interval)*
Pre-Implementation Trend	-5.91[d] (-6.77, -5.04)	-13.21[d] (-14.44, -11.98)	-0.90[d] (-0.99, -0.81)	-1.25[d] (-1.35, -1.15)
Quarter of Implementation	-26.11[d] (-36.75, -15.46)	-36.06[d] (-51.22, -20.90)	-3.64[d] (-4.91, -2.37)	-4.49[d] (-5.87, -3.10)
1 Quarter After Implementation	-34.46[d] (-45.54, -23.39)	-36.74[d] (-52.51, -20.97)	-4.02[d] (-5.34, -2.70)	-4.20[d] (-5.64, -2.76)
2 Quarters After Implementation	-35.29[d] (-46.15, -24.43)	(-48.30[d] (-63.77, -32.83)	-4.18[d] (-5.47, -2.88)	-5.61[d] (-7.01, -4.20)
3 Quarters After Implementation	-39.96[d] (-51.59, -28.34)	-44.91[d] (-61.47, -28.35)	-4.60[d] (-5.94, -3.25)	-6.44[d] (-7.91, -4.97)
4 Quarters After Implementation	-27.60[d] (-39.37, -15.83)	-28.60[c] (-45.37, -11.84)	-3.11[d] (-4.46, -1.77)	-5.04[d] (-6.51, -3.58)
5 Quarters After Implementation	-15.61 (-32.01, 0.78)	-3.05 (-26.40, 0.30)	-1.07 (-2.96, 0.82)	-2.59[b] (-4.64, -0.53)

[a]Interrupted time series analysis done using quantile regression of MME and tablet number using procedure type as a fixed effect
[b]p<0.05 [c]p<0.01 [d]p<0.001

eTable 3. Interrupted Time Series Regression Analysis Results By Type of Surgery[a]

	Mean MME[a]	Median MME[b]	Mean Tabs[a]	Median Tabs[b]
	**A. Laparoscopic Cholecystectomy**			
	Coefficient (95% Confidence Interval)	*Coefficient (95% Confidence Interval)*	*Coefficient (95% Confidence Interval)*	*Coefficient (95% Confidence Interval)*
Pre-Implementation Trend	-7.94[e] (-10.17, -5.71)	-5.55[e] (-5.85, -5.25)	-0.90[e] (-1.18, -0.62)	0 (-0.07, 0.07)
Quarter of Implementation	-15.29[e] (-28.32, -2.26)	-33.33[e] (-38.48, -28.19)	-3.93[e] (-5.88, -1.99)	-10.00[e] (-11.17, -8.82)
1 Quarter After Implementation	-8.46 (-22.71, 5.79)	-27.78[e] (-33.21, -22.35)	-3.24[d] (-5.37, -1.11)	-10.00[e] (-11.24, -8.76)
2 Quarters After Implementation	-0.46 (-16.06, 15.13)	-22.22[e] (-27.76, -16.69)	-2.88[d] (-5.69, -0.08)	-10.00[e] (-11.26, -8.74)
3 Quarters After Implementation	3.45 (-17.05, 23.95)	-16.67[e] (-22.18, 11.15)	-3.06[c] (-4.82, -0.04)	-10.00[e] (-11.26, -8.74)
4 Quarters After Implementation	2.73 (-18.70, 24.16)	-11.11[e] (-16.78, -5.44)	-3.06[c] (-6.03, -0.10)	-10.00[e] (-11.29, -8.70)
5 Quarters After Implementation	15.83 (-9.34, 41.00)	-5.55 (-14.51, 3.40	-1.21 (-4.67, -2.25)	-10.00[e] (-12.05, -7.96)
	**B. Cesarean Section**			
	Mean MME[a]	Median MME[b]	Mean Tabs[a]	Median Tabs[b]
	Coefficient (95% Confidence Interval)	*Coefficient (95% Confidence Interval)*	*Coefficient (95% Confidence Interval)*	*Coefficient (95% Confidence Interval)*
Pre-Implementation Trend	-3.89[e] (-5.05, -2.73)	0 (-0.59, 0.59)	-0.67[e] (-0.79, -0.54)	0 (-0.01, 0.01)
Quarter of Implementation	-21.13[e] (-32.38, -9.88)	0 (-9.97, 9.97)	-4.60[e] (-6.00, -3.20)	-10.00[e] (-10.10, -9.90)
1 Quarter After Implementation	-24.04[e] (-35.63, -12.46)	0 (-10.27, 10.27)	-5.27[e] (-6.66, -3.87)	-10.00[e] (-10.10, -9.90)
2 Quarters After Implementation	-22.40[e] (-33.48, -11.32)	0 (-10.90, 10.90)	-5.28[e] (-6.68, -3.88)	-10.00[e] (-10.10, -9.89)
3 Quarters After Implementation	-18.79[d] (-30.56, -7.01)	-10.00 (-20.77, 0.77)	-4.93[e] (-6.47, -3.39)	-10.00[e] (-10.10, -9.90)
4 Quarters After Implementation	-8.92 (-23.30, 5.45)	0 (-11.46, 11.46)	-3.84[e] (-5.76, -1.91)	-10.00[e] (-10.11, -9.89)
5 Quarters After Implementation	-3.27 (-20.51, 13.97)	-10.00 (-27.66, 7.66)	-3.36[d] (-5.65, -1.06)	-10.00[e] (-10.17, -9.83)
	**C. Knee Arthroscopy**			
	Mean MME[a]	Median MME[b]	Mean Tabs[a]	Median Tabs[b]
	Coefficient (95% Confidence Interval)	*Coefficient (95% Confidence Interval)*	*Coefficient (95% Confidence Interval)*	*Coefficient (95% Confidence Interval)*
Pre-Implementation Trend	-10.30[d] (-16.19, -4.41)	-10.00[e] (-11.97, -8.03)	-1.00[d] (-1.61, -0.39)	-0.83 (-1.09, -0.58)
Quarter of Implementation	-5.75 (-48.49, -37.00)	10.00 (-22.33, 42.33)	-2.29 (-6.36, 1.78)	0.83 (-3.39, 5.05)
1 Quarter After Implementation	4.13 (-50.33, 58.59)	20.00 (-16.00, 56.00)	-1.07 (-6.04, 3.90)	1.67 (-3.03, 6.36)
2 Quarters After Implementation	-17.69 (-76.41, 41.02)	30.00 (-6.60, 66.60)	-3.79 (-10.05, 2.46)	2.50 (-2.28, 7.28)

© 2021 Shenoy R et al. *JAMA Health Forum*.

3 Quarters After Implementation	-1.51 (-54.22, 51.20)	40.00[c] (1.74, 78.26)	-1.73 (-6.48, 3.02)	3.33 (-1.66, -8.33)
4 Quarters After Implementation	21.64 (-28.03, 71.32)	50.00[d] (12.83, 87.17)	-0.52 (-5.51, 4.46)	4.17 (-0.69, 9.02)
5 Quarters After Implementation	15.58 (-42.86, 74.02)	60.00[c] (4.49, 115.51)	-0.36 (-6.08, 5.36)	5.00 (-2.25, 12.25)

[a] Interrupted time series analysis done using linear regression of mean MME, clustering standard errors by prescribing provider
[b]Interrupted time series analysis done using quantile regression of MME (medians shown here)
[c]p<0.05, [d]p<0.01, [e]p<0.001

eTable 4. Inputs for Calculation of Tablet Reduction[a]

	General			OB/GYN			Ortho		
	Procedures	Effect	Tablet Reduction	Procedures	Effect	Tablet Reduction	Procedures	Effect	Tablet Reduction
Quarter 0	2,442	-3.07	-7,505.87	1,438	-4.63	-6,661.22	1,207	-4.07	-4,917.37
Quarter 1	2,447	-2.33	-5,704.05	1,409	-5.07	-7,149.80	1,234	-4.11	-5,073.82
Quarter 2	2,504	-3.18	-7,972.65	1,292	-5.14	-6,641.81	1,287	-4.91	-6,314.12
Quarter 3	2,653	-2.75	-7,287.74	1,437	-4.88	-7,011.73	1,162	-5.54	-6,435.48
Quarter 4	2,789	-2.43	-6,781.86	1,299	-3.87	-5,023.48	1,368	-4.10	-5,606.56
Quarter 5	959	-2.26	-2,162.60	433	-3.36	-1,456.61	489	-1.84	-901.91
		**Total**	**-37,415**		**Total**	**-33,945**		**Total**	**-29,249**
**Total Fewer Pills**	**-100,609**								

[a]Number of procedures is based on total procedures in the quarters after the CURES mandate was implemented. Effects are based on the mean tablet reductions from table 2. Reductions are based in the effect multiplied by the number of procedures.

© 2021 Shenoy R et al. *JAMA Health Forum*.

eTable 5. Interrupted Time Series Regression Analysis for 5-Day Duration

eTable *. Proportion of Prescriptions Exceeding a Five-Day Supply Decreased at the time of CURES Mandate: Interrupted Time Series Regression Analysis			
	General Surgery	Obstetrics/Gynecologic Surgery	Orthopedic Surgery
	Coefficient (95% Confidence Interval)	*Coefficient (95% Confidence Interval)*	*Coefficient (95% Confidence Interval)*
Pre-Implementation Trend	-0.10[d] (-0.11, -0.10)	-0.07[d] (-0.08, -0.06)	-0.08[d] (-0.10, -0.07)
Quarter of Implementation	-0.53[d] (-0.65, -0.40)	-0.58[d] (-0.75, -0.41)	-0.42[d] (-0.59, -0.25)
1 Quarter After Implementation	-0.44[d] (-0.55, -0.32)	-0.48[d] (-0.65, -0.31)	-0.62[d] (-0.80, -0.45)
2 Quarters After Implementation	-0.71[d] (-0.84, -0.58)	-0.69[d] (-0.88, -0.50)	-0.69[d] (-0.87, -0.51)
3 Quarters After Implementation	-0.73[d] (-0.87, -0.60)	-0.36[d] (-0.54, -0.19)	-0.82[d] (-1.01, -0.63)
4 Quarters After Implementation	-0.70[d] (-0.83, -0.56)	-0.53[d] (-0.72, -0.34)	-0.72[d] (-0.91, -0.52)
5 Quarters After Implementation	-0.73[d] (-0.95, -0.52)	-0.81[d] (-1.14, -0.48)	-0.37[c] (-0.64, -0.10)

[a]Interrupted time series analysis done using logistic regression, using procedure type as a fixed effect
[b]p<0.05, [c]p<0.01, [d]p<0.001

© 2021 Shenoy R et al. *JAMA Health Forum*.

eFigure 1. Patient Flowchart and Exclusion Criteria Rationale

eFigure 1: Patient Flowsheet and Exclusion Criteria Rationale

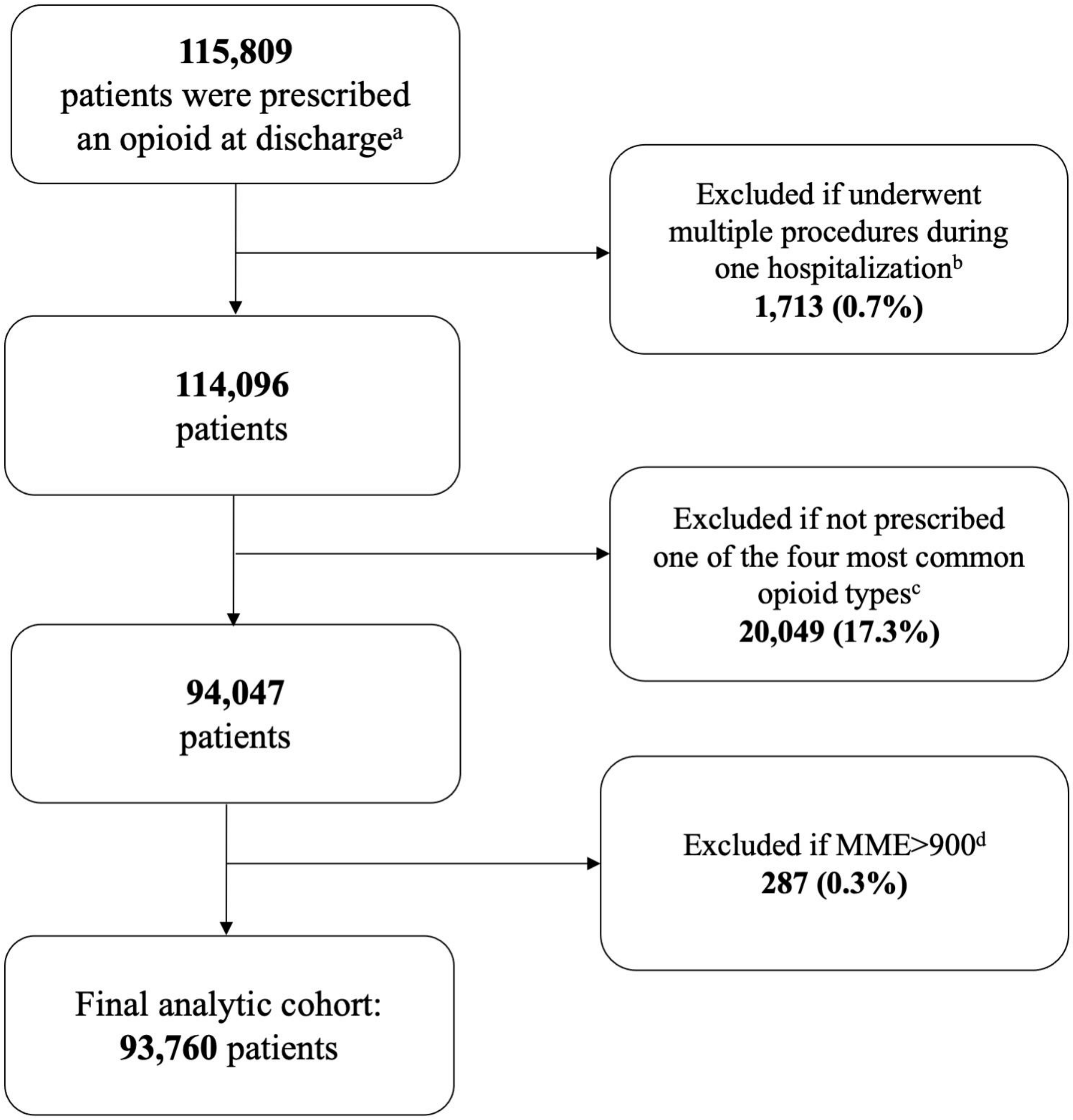

Abbreviations: MME, Morphine Milligram Equivalents

[a]Within general, orthopedic and obstetric and gynecologic service lines

[b]Exclusion applied given that patients undergoing multiple procedures may require higher discharge Prescriptions and comparison to those only receiving one procedure may not be appropriate.

[c]Exclusion applied in order to compare tablet number prescribed with evidence-based guidelines that use oxycodone 5mg as a reference. Also included the most common opioid types if more commonly prescribed than oxycodone 5mg.

[d]Exclusion applied in order to rule out any erroneously placed prescriptions, or prescriptions for patients who may require significantly higher post-operative opiates for an unidentified reason.

eFigure 2. Distribution of Morphine Milligram Equivalents and Number of Tablets

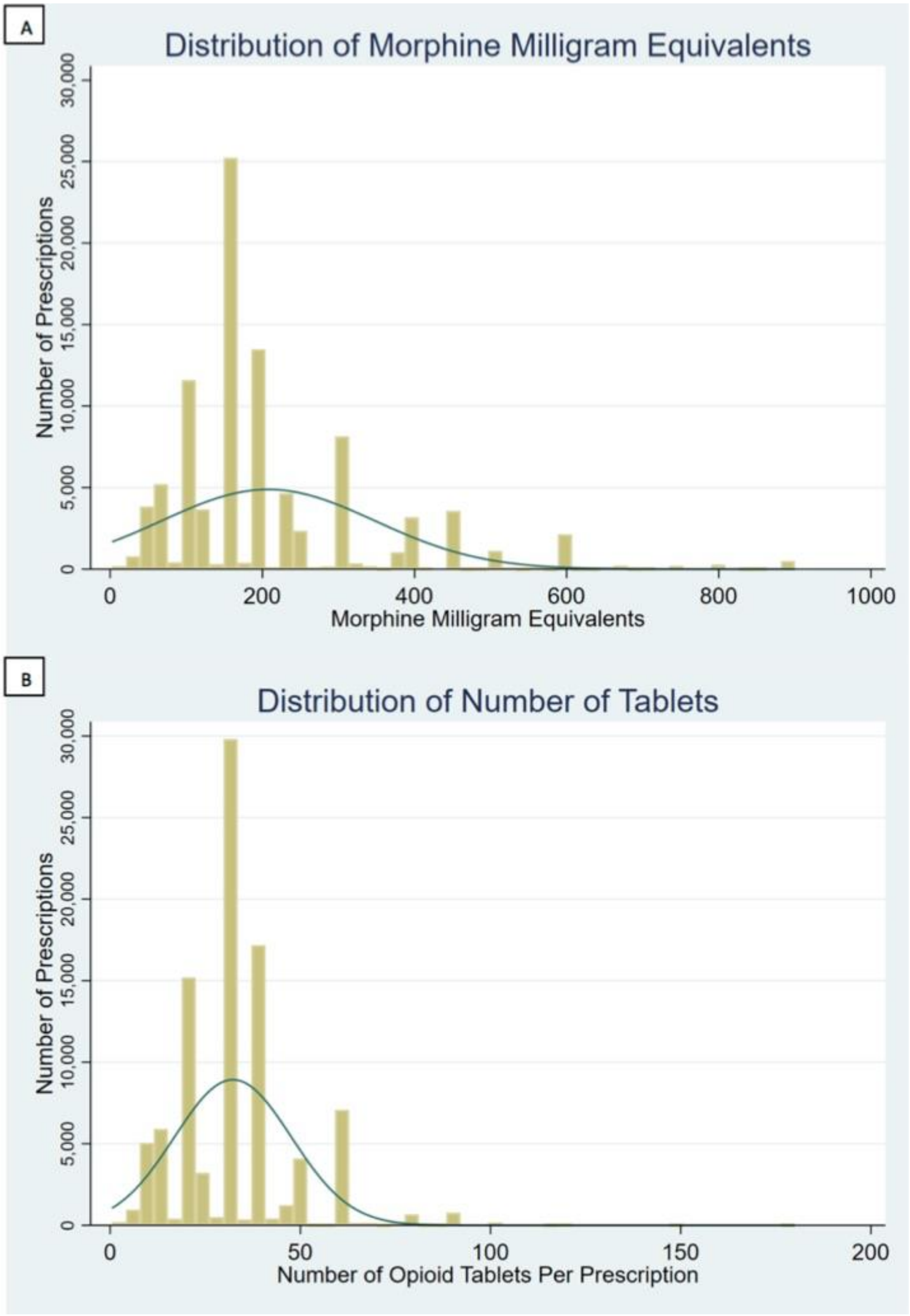

© 2021 Shenoy R et al. *JAMA Health Forum*.

eFigure 3. Change in Opioid Type Prescribed for Cesarean Delivery at Time of CURES Mandate Implementation

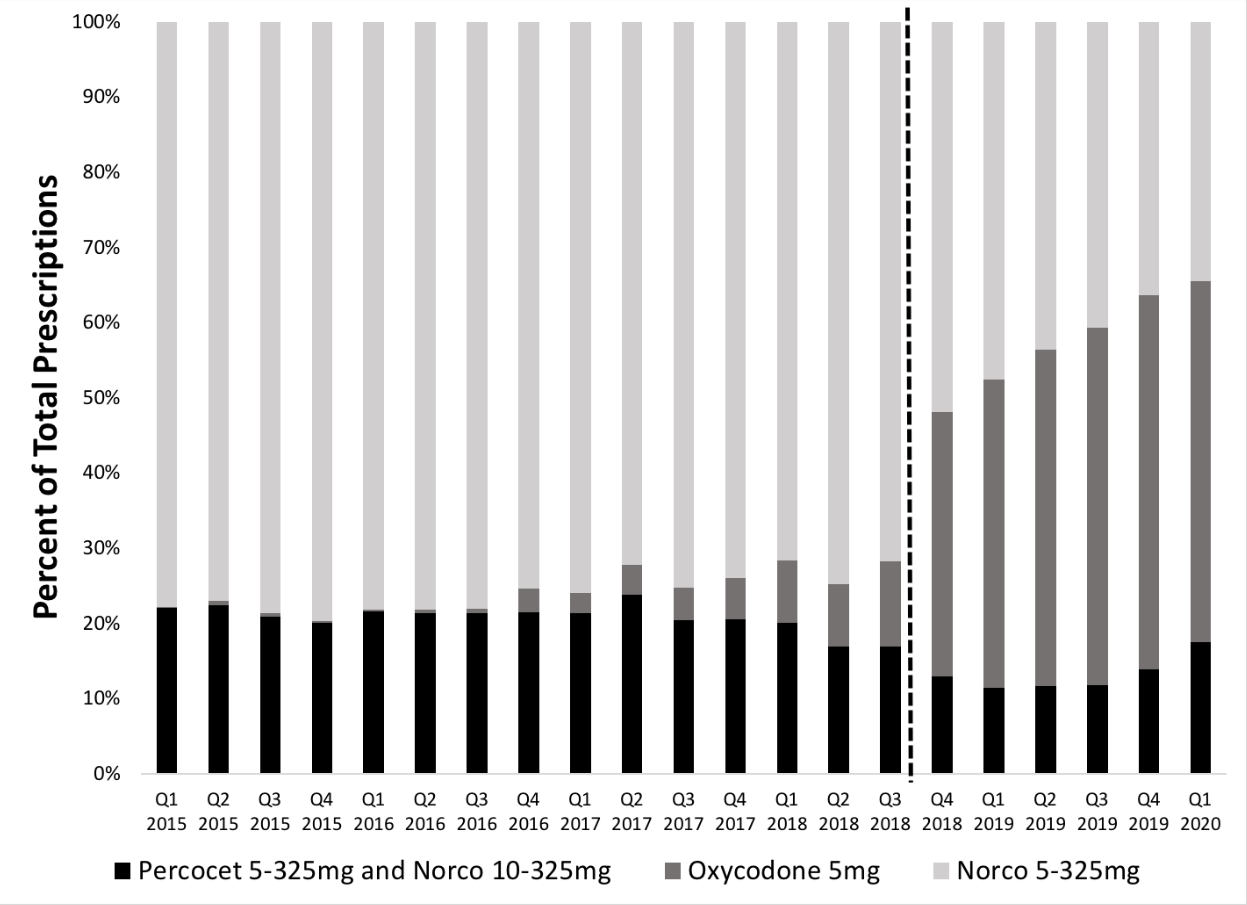

© 2021 Shenoy R et al. *JAMA Health Forum*.

eFigure 4. Patterns in Prescriptions With a Greater Than 5-Day Duration

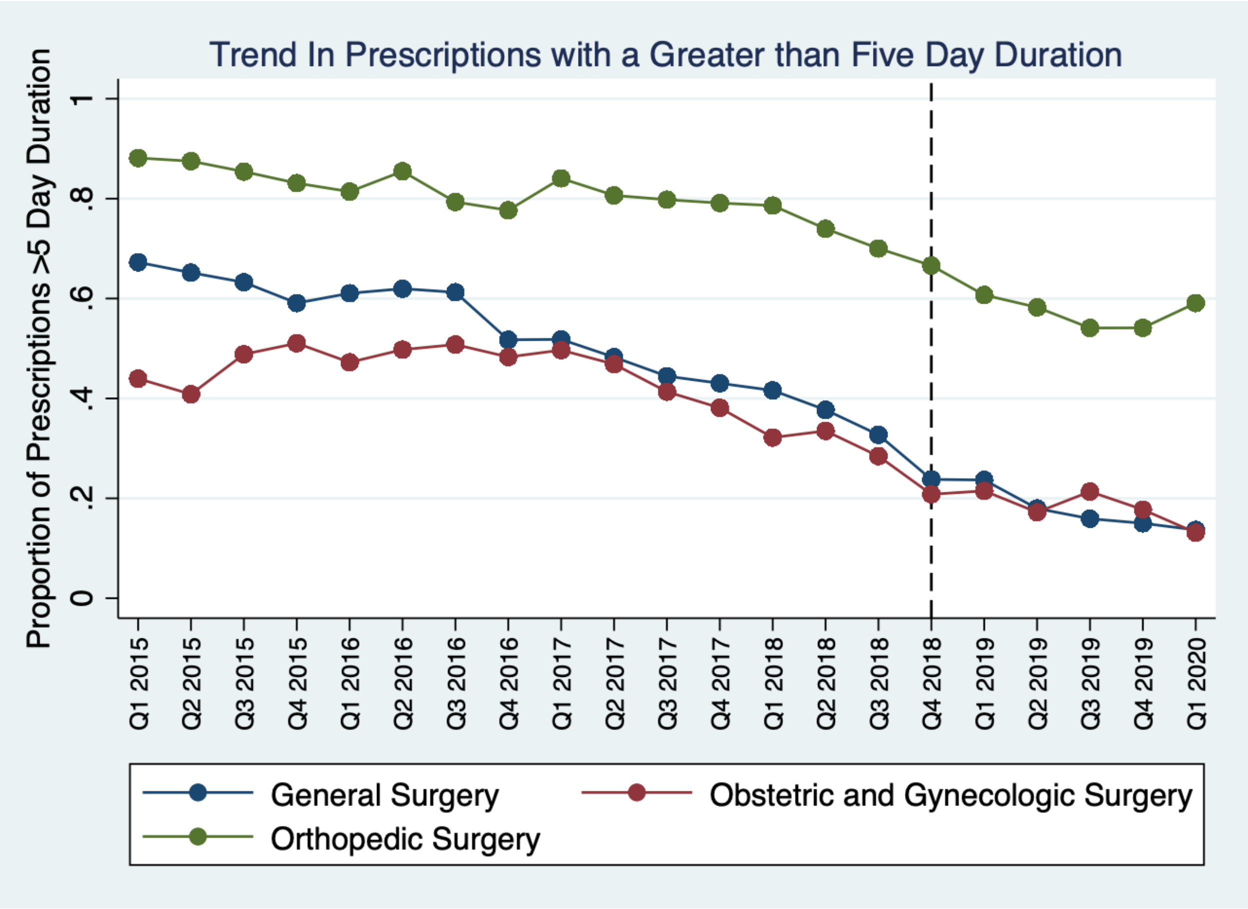

© 2021 Shenoy R et al. *JAMA Health Forum*.
